# Pelvic Outlet Obstruction Secondary to Salmonella enterica serovar Bovismorbificans Abscess

**DOI:** 10.7759/cureus.12865

**Published:** 2021-01-22

**Authors:** Riley A Scanlan, Katherine E Kramme, Kristofer E Nava, Shaan Manawar, Saad Shebrain

**Affiliations:** 1 Surgery, Western Michigan University Homer Stryker M.D. School of Medicine, Kalamazoo, USA

**Keywords:** salmonella enterica serovar bovismorbificans, pelvic outlet obstruction, intra-abdominal abscess

## Abstract

We present a case of a 30-year-old Hispanic male with pelvic outlet obstruction syndrome secondary to a large pelvic abscess caused by *Salmonella* *enterica* Bovismorbificans. This case demonstrates a potentially serious complication of a rare foodborne illness in the United States, in which an urgent surgical intervention was warranted. A computed tomography (CT) scan of the abdomen and pelvis demonstrated a large pelvic cystic mass causing near-total pelvic outlet obstruction of both gastrointestinal and genitourinary systems. A total of 1,250 mg of IV vancomycin and 3.375 mg of IV piperacillin-tazobactam were administered every eight hours, and an urgent decompressive transverse loop colostomy, Foley catheter placement, and percutaneous drainage were performed. Culture of the abscess fluid identified *Salmonella* *enterica* serotype Bovismorbificans, and the antibiotic regimen was changed to 1,000 mg IV ceftriaxone every 24 hours. Subsequent CT imaging displayed a reduction in abscess size. The patient was then discharged with a 14-day course of 500 mg of oral ciprofloxacin every 12 hours and 500 mg of oral metronidazole every eight hours. Imaging at three weeks post-discharge displayed resolution of the abscess, and the drain was removed. The patient had complete recovery and did well several months following treatment. While rare, *Salmonella* *enterica* serotype Bovismorbificans could potentially lead to serious complications such as giant pelvic abscess, in which a multidisciplinary team approach (i.e., medical, surgical, and interventional) is critical for a good outcome.

## Introduction

*Salmonella enterica* is a gram-negative bacillus commonly implicated in foodborne illnesses in the United States. It is generally transmitted by the consumption of eggs, chicken, and pork, as well as various other foods including vegetables and other produce. The most common serotypes are *Enteritidis*, *Typhimurium*, *Newport*, and *Heidelberg*, which account for 66% of Salmonella outbreaks [1]. These infections generally result in gastroenteritis of a varying severity, with most patients experiencing some degree of nausea, vomiting, diarrhea, abdominal cramps, and low-grade fever.

*Salmonella enterica* serotype Bovismorbificans, however, is encountered much more rarely and has only been implicated in five outbreaks in the United States since 2001 [2], in which case, hummus, tahini, alfalfa sprouts, cheese, pasta salad, and striped bass were identified as sources. These outbreaks were associated with similar symptoms as the other more common Salmonella serotypes, and no cases resulted in the subsequent development of pelvic abscess or pelvic outlet obstruction syndrome. As such, we present a rare case of *S. enterica* ser. Bovismorbificans involving a clinical presentation previously undescribed by the current medical literature. There have been no previous publications indicating the ability of this bacterium to cause an abscess of such magnitude to result in obstruction of the pelvic outlet. Therefore, this case offers an opportunity to observe a rare sequela of *S. enterica* infection.

## Case presentation

The patient was a 30-year-old male agriculture worker with a history of diabetes mellitus requiring insulin and without past surgical history who presented to the Emergency Department (ED) with abdominal pain, progressive abdominal distention, constipation, urine retention, and swelling in the right lower extremity. Physical examination demonstrated a temperature of 101.3°F, heart rate of 113 beats per minute, and respiratory rate of 22 breaths per minute. Abdominal examination showed no previous scars but revealed moderate distention and significant tenderness in the epigastric region and in the right and left lower quadrants. Right lower extremity showed non-pitting edema. The patient denied any family history of gastrointestinal illness upon questioning and denied any similar past medical history. Laboratory tests showed severe hyperglycemia without ketosis (glucose of 788 mg/dL, anion gap of 15 mEq/L), hyponatremia (sodium of 117 mEq/L, corrected sodium of 128 mEq/L), elevated creatinine (1.6 mg/dL), mildly elevated alkaline phosphatase (210 IU/L), and elevated acute phase reactants (C-reactive protein of 15.1 mg/dL, pro-calcitonin of 1.34 ng/mL). White blood cells were within normal limits (9.7 x 10^9^/L), and blood cultures were negative after 72 hours.

**Figure 1:**
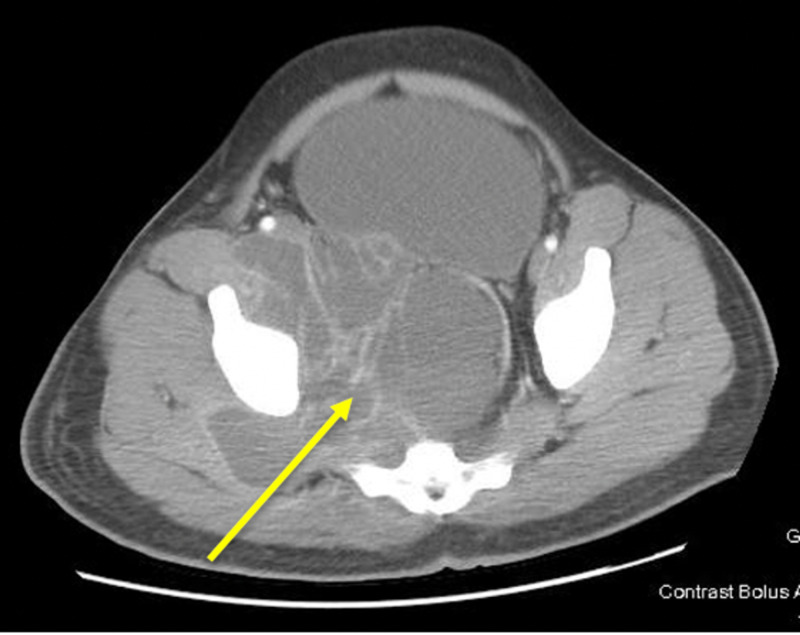
Transverse CT section demonstrating a 14 x 17 cm cystic mass with extra-pelvic extension (yellow arrow).

**Figure 2:**
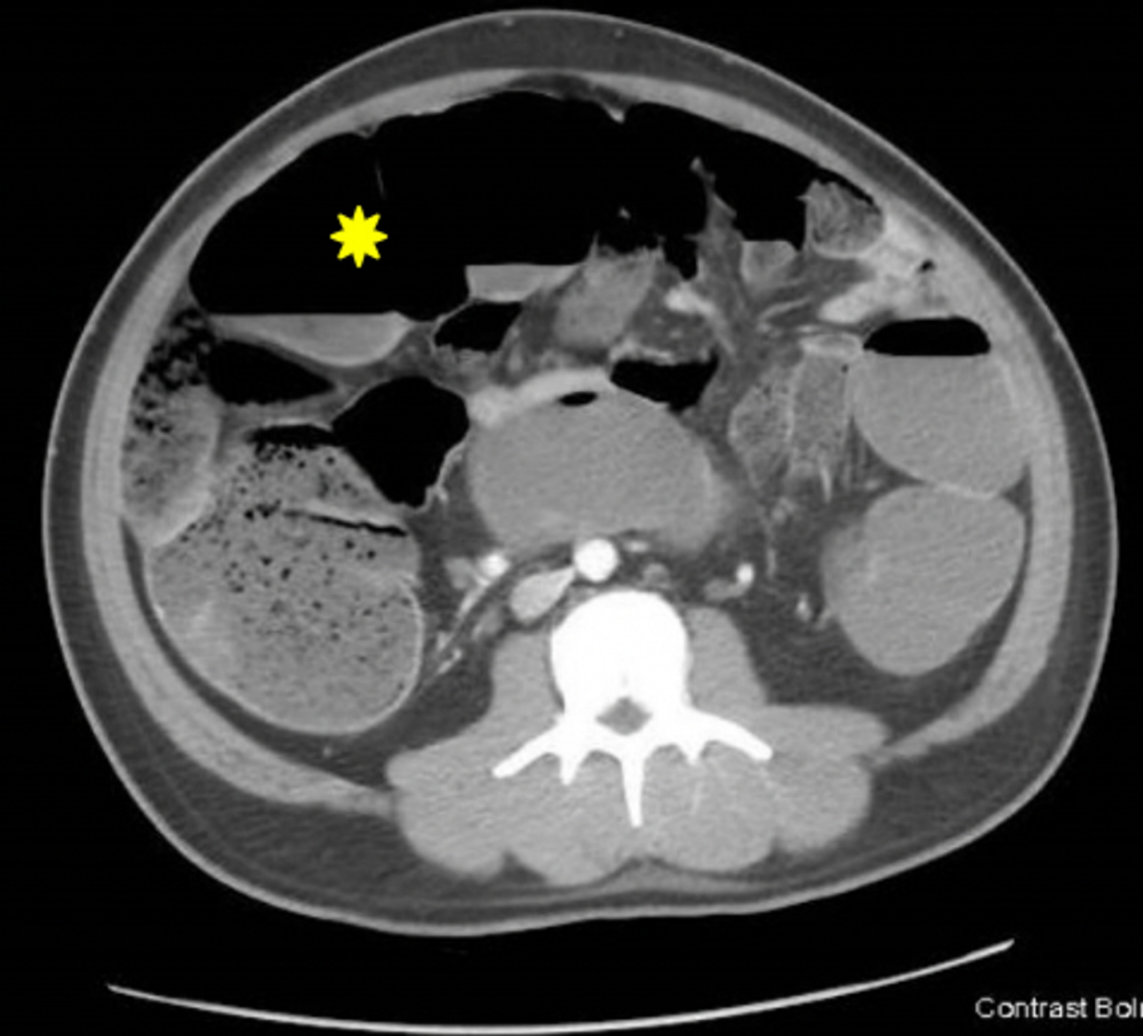
Transverse CT section demonstrating large bowel obstruction (yellow asterisk).

**Figure 3:**
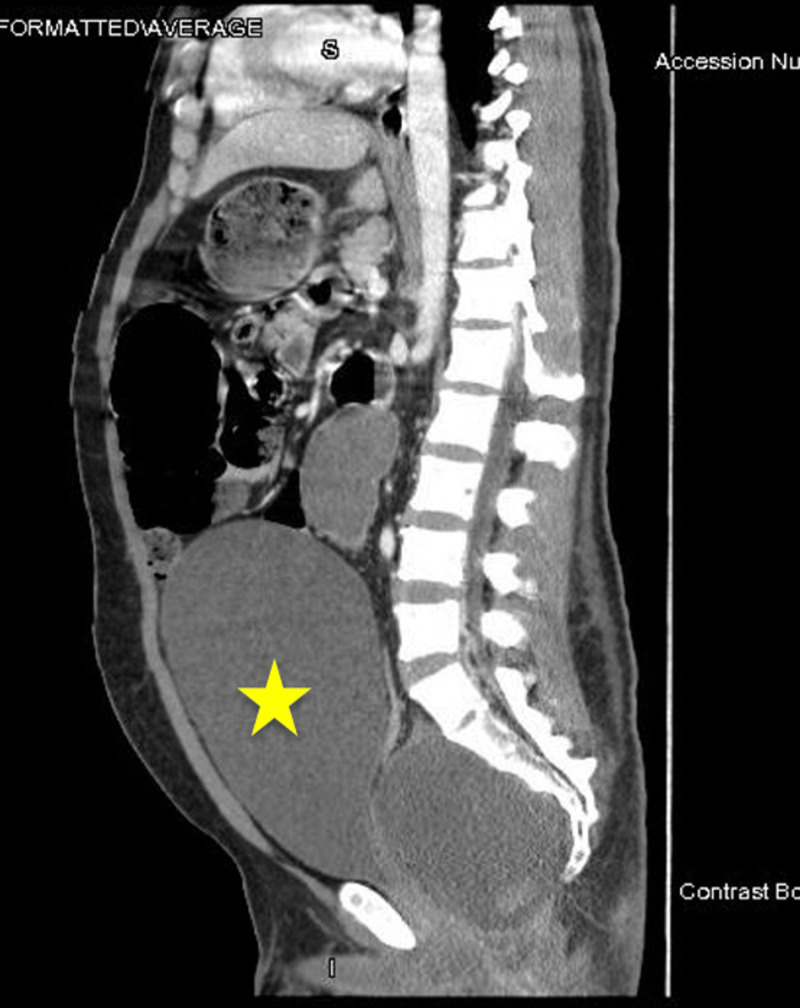
Severe obstructive uropathy (yellow star) involving the bladder with bilateral obstructive hydronephrosis.

An initial intravenous (IV) bolus of balanced crystalloid fluid was given, and a CT scan of the abdomen and pelvis was obtained (Figures 1-3). It showed a multiseptated pelvic mass/cystic lesion measuring 14 cm by 17 cm, which extended to the right inguinal canal anteriorly and the right gluteal muscle posteriorly. There was a significant mass effect with near-total pelvic outlet obstruction affecting both gastrointestinal and genitourinary tracts as manifested by large intestine obstruction near the rectosigmoid junction and obstructive uropathy with hydronephrosis.

Given the clinical findings and concerns for abdominal catastrophe, the patient was initially given a bolus of 1,000 mL of 0.9 NaCl fluid before obtaining the CT scan. With the CT findings indicating a large infected fluid and the existence of hyperglycemia, the patient received an additional 3 L of normal saline with insulin drip for a short period. However, the patient's blood sugar normalized over the next 24 hours once the infection source was controlled.

**Figure 4:**
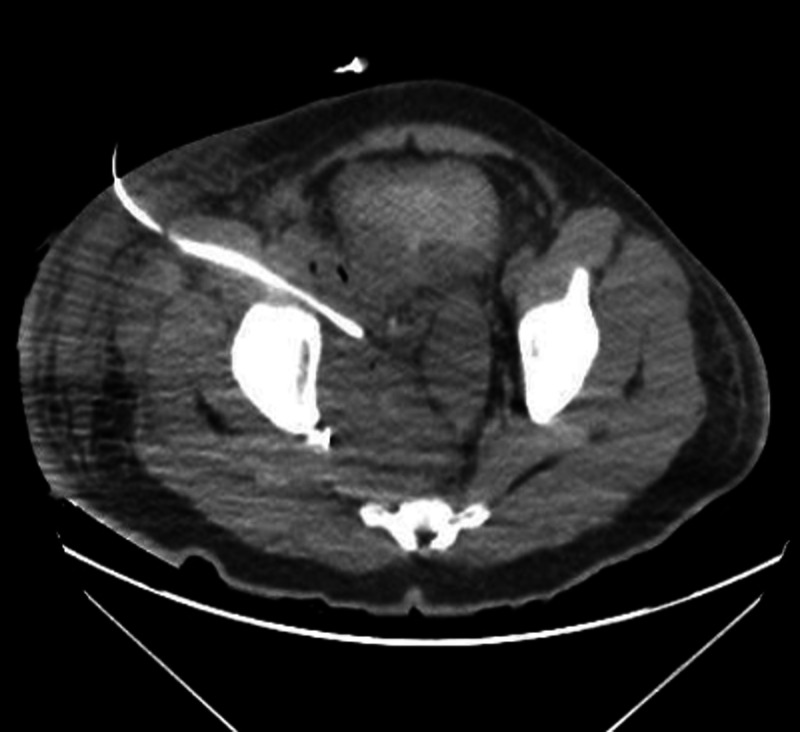
The seven-day postoperative CT scan (transverse section) displaying partial resolution of the pelvic abscess.

In addition to IV fluids, empirical broad-spectrum antibiotics (1,250 mg of IV vancomycin and 3.375 mg of IV piperacillin-tazobactam every eight hours) were initiated, and a nasogastric tube was inserted for gastric decompression. A Foley catheter was placed, and an immediate return of 1,000 mL of urine was observed. Given the degree of large bowel obstruction and the presence of abdominal pain, the patient was immediately transported to an operating room where a decompressive transverse loop colostomy was performed. Postoperatively, a CT-guided drainage of the abscess was performed, and an initial 500 mL of purulent fluid of a red/brown color was retrieved. Fluid analysis was performed including cytology. Results were negative for malignancy. Fluid cultures, however, were positive for *S. enterica* stereotype Bovismorbificans, and susceptibility testing revealed sensitivity to ceftriaxone (minimum inhibitory concentration ≤ 1 µg/mL) and ciprofloxacin (minimum inhibitory concentration ≤ 0.12 µg/mL). In light of this identification and to implement proper antibiotic stewardship procedures, the patient was switched from IV vancomycin and piperacillin-tazobactam to 1,000 mg of IV ceftriaxone every 24 hours. Seven days postoperatively, a CT scan of the abdomen and pelvis was obtained (Figure 4), which showed a significant reduction in the size of the pelvic abscess. However, there was a residual abscess and, therefore, a second drain was inserted under CT guidance.

**Figure 5:**
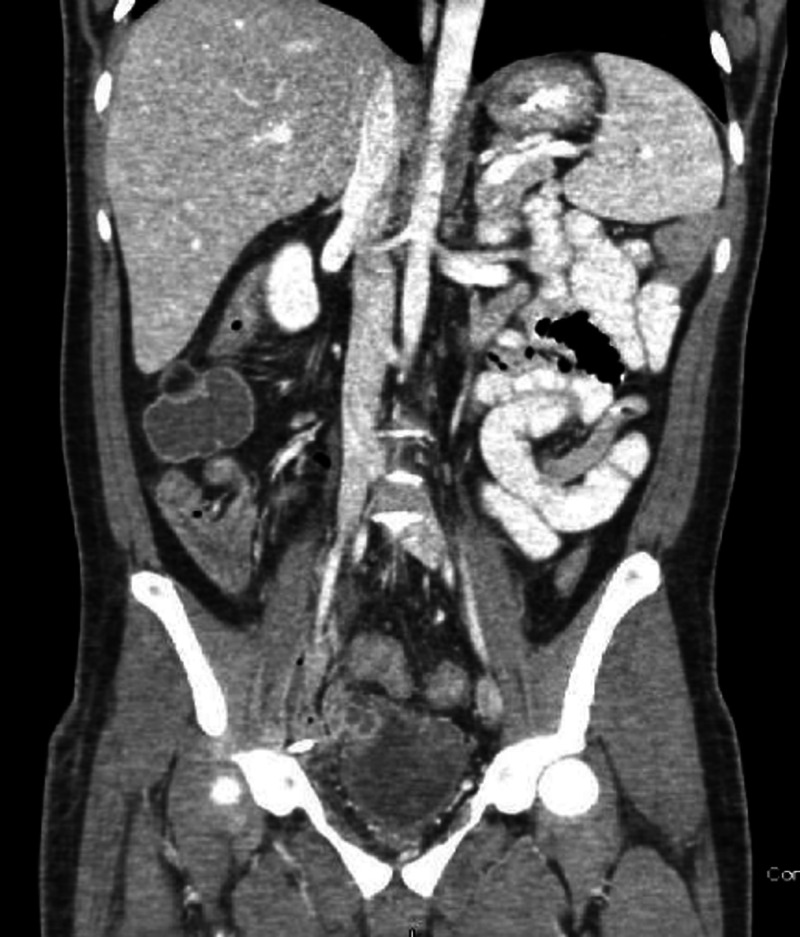
The 21-day postoperative CT scan (coronal section) showing abscess resolution. Drainage catheters have also been removed.

On postoperative day 10, the patient was discharged from the hospital to home with an additional 14-day course of 500 mg of oral ciprofloxacin to be taken every 12 hours and 500 mg of oral metronidazole to be taken every eight hours. A three-week post-discharge follow-up CT scan revealed a complete resolution of the pelvic abscess (Figure 5). A tube check for the two drains showed minimal drainage with no evidence of any fistulation or residual cavities, and, therefore, they were discontinued.

**Figure 6:**
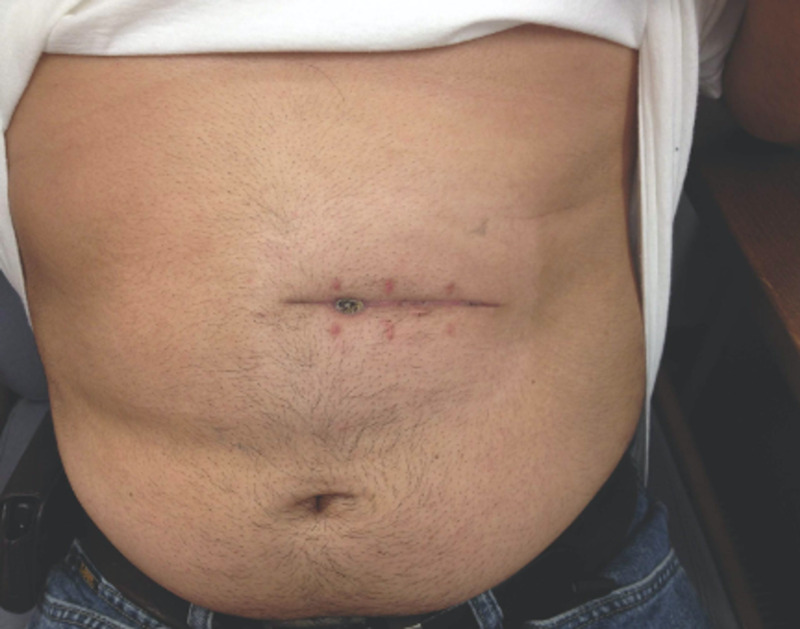
Wound healing two weeks after colostomy reversal.

Four months following colostomy creation and before colostomy reversal was performed, the patient underwent a preoperative colonoscopic examination, which was negative for any colonic lesions secondary to *Salmonella* infection. A two-week postoperative follow-up showed appropriate healing of surgical incision with no persistence or recurrence of original presenting symptoms (Figure 6).

## Discussion

*Salmonella* is a facultative intracellular, anaerobic, gram-negative, rod-shaped bacillus and is one of the most common causes of gastrointestinal illness in the United States [3]. This pathogen is commonly associated with foodborne outbreaks, is responsible for over 23,000 hospital admissions each year, and causes over 400 annual fatalities [4]. Additionally, *Salmonella* can be characterized by its ability to cause typhoid fever as a result of septic infection [5]. Most cases of gastroenteritis are caused by infection with the more common serotypes discussed previously. *Salmonella* *enterica* serotype Bovismorbificans, however, is rarely associated with foodborne outbreaks. When outbreaks do occur, they are associated with a wide variety of foods and locations, including hummus, tahini, ham, and alfalfa seeds [1,6,7]. Between the years 1999 and 2009, despite over 400,000 cases of salmonellosis in the United States, only 758 were a result of Bovismorbificans [8]. Another study conducted during the period of 2006-2016 showed 817 cases of S. Bovismorbificans throughout the United States compared to 83,303 cases of *S. Enteritidis*, 63,773 cases of *S. Typhimurium*, and 47,481 cases of *S. Newport* [9].

In most cases, *Salmonella* species present with gastroenteritis, but, less commonly, infection may develop into intra-abdominal infection and abscess formation, with localized abscesses forming in the spleen and the ovary, as documented in previously published case reports [10,11]. Additionally, soft tissue abscesses have also been documented in some case reports [12]. No randomized controlled trials have been published that have studied this rare presentation. Importantly, no cases of intra-abdominal abscess secondary to S. *enterica* Bovismorbificans have been published. As such, the presentation of pelvic outlet obstruction resulting from *Salmonella*-induced intra-abdominal abscess formation is extremely rare.

Because of this rarity, the less common sequelae of the already rare serotype Bovismorbificans are poorly understood and unlikely to be anticipated by clinicians in the case of *Salmonella* infection. This case, however, demonstrates the ability of the stereotype Bovismorbificans to cause massive intra-abdominal and pelvic abscesses capable of creating a pelvic outlet obstruction, a sequela previously undescribed in the current body of literature. Because of the potential severity of this complication, the clinician should be aware that although the occurrence of Bovismorbificans infections is relatively rare, a high index of suspicion is important when evaluating the differential diagnosis of intra-abdominal and pelvic abscess, especially in cases of co-existing gastroenteritis symptoms.

The pathophysiology underlying the development of intra-abdominal abscess following *Salmonella* infection is poorly understood, but it is likely that the gastroenteritis secondary to the infection resulted in an area of localized inflammation that precipitated the development of the intra-abdominal abscess. Unfortunately, neither colonoscopy nor CT imaging provided evidence of the original source of the abscess, and without additional cases to compare, it is unknown whether a certain region of the gastrointestinal tract is more likely to be the source of an abscess. Additionally, it is not well understood if certain serotypes of *Salmonella* display a propensity for abscess formation. If this is the case, it is possible that these serotypes, such as Bovismorbificans, possess structural or functional differences that prevent effective clearance of the pathogen and improve its ability to migrate to the intra-abdominal cavity. Even so, most affected patients do not develop abscess, meaning that other factors, such as environmental triggers, changes in the microbiome, or genetic variability, may increase the risk of abscess development.

Due to the limited scope of this case report and the lack of controlled trials on the subject of Bovismorbificans infection, more research would be necessary to determine if serotype testing for Bovismorbificans or other high-risk serotypes could potentially alter screening and treatment decisions. Such research could focus on the structural or functional differences that prevent effective clearance of the pathogen and improve its ability to migrate to the intra-abdominal cavity. If Bovismorbificans represents a greater risk for abscess development compared to other *Salmonella* species, then serotyping *Salmonella* outbreaks and even isolated *Salmonella* cases would be warranted. Even with the lack of randomized trial evidence, the clinician should be careful to consider this sequela when treating suspected *Salmonella* cases. In addition, more research needs to be conducted to determine if patient demographics would indicate a higher risk of intra-abdominal abscess. Again, this would allow the clinician to gauge the level of risk for certain patients and tailor treatment decisions based on that risk.

## Conclusions

*Salmonella enterica* serotype Bovismorbificans is a rare cause of infection and most commonly presents with gastroenteritis in the setting of foodborne outbreaks. However, rarely Bovismorbificans can present with acute surgical emergency due to massive intra-abdominal abscess causing obstructive symptoms even without exposure to known sources. A timely multidisciplinary approach including surgery, image-guided intervention, and antibiotic therapy is critical for achieving a good outcome. Importantly, a high index of suspicion of this rare complication is important and should be part of the differential diagnosis in order to prevent unnecessary complications.
